# AI-generated images of familiar faces are indistinguishable from real photographs

**DOI:** 10.1186/s41235-025-00683-w

**Published:** 2025-10-14

**Authors:** Robin S. S. Kramer, Alex L. Jones, Daniel Fitousi, Jeremy J. Tree

**Affiliations:** 1 School of Psychology, Sport Science & Wellbeing, University of Lincoln, Lincoln, UK; 2 School of Psychology, Swansea University, Swansea, UK; 3 Department of Psychology, Ariel University, Ariel, Israel

**Keywords:** Face perception, Deepfakes, ChatGPT, Artificial intelligence

## Abstract

Human users are now able to generate synthetic face images with artificial intelligence (AI) tools. Although indistinguishable from real photographs, these images have tended to feature fictional identities that do not exist in the real world. As a result, their use in applied contexts, including the spread of fake information, is similarly limited. Here, we investigated a new method for generating face images (via ChatGPT plus DALL·E) and its application to both fictional and real (in this case, celebrity) identities. Our results demonstrated that generated images of both fictional (Experiment 1) and celebrity identities (Experiment 2) could not be distinguished from real photographs. Further, providing additional real photographs for comparison during the task resulted in limited gains (Experiments 3 and 4). Finally, prior familiarity with celebrity faces produced only modest performance improvements. Therefore, new methods of detection should be explored as a matter of urgency since the latest ‘off the shelf’ AI tools can now generate face images of real people that are essentially undetectable as synthetic to most human observers.

**Supplementary Information:**

The online version contains supplementary material available at 10.1186/s41235-025-00683-w.

## Introduction

Early attempts to create synthetic human faces in domains including robotics and entertainment tended to lack realism, often falling into the “uncanny valley” (Mori, 1970/2012) and eliciting unease or repulsion in observers (for reviews, see Kätsyri et al., 2015; Wang et al., 2015). More recently, advances in artificial intelligence (AI) have resulted in techniques that are now capable of traversing this valley successfully. For instance, images synthesised using generative adversarial networks (GANs) like StyleGAN2 (Karras et al., 2020, 2021) are indistinguishable from, and often more realistic than, real face photographs (e.g. Bray et al., 2023; Kramer & Cartledge, 2024; Lago et al., 2022; Miller et al., 2023; Nightingale & Farid, 2022; Tucciarelli et al., 2022). Problematically, such images are now being used in fake social media profiles (e.g. Ricker et al., 2024) with the possibility of malicious intent (e.g. to influence public opinion or political behaviour).

To date, generating synthetic face images has been limited to new identities (i.e. ‘people’ who do not exist). Acceptance as genuine requires ‘only’ that these faces achieve anatomical realism and photographic quality. Beyond this, viewers have no expectations regarding how each face should look. In contrast, producing synthetic images of familiar faces represents a more challenging task by incorporating a further requirement – the image must fall within the boundaries of plausibility (based on prior knowledge; Burton et al., 2016) for that specific person. Previous research has shown that viewers are sensitive to even small alterations to familiar face images (e.g. Brédart & Devue, 2006; Brooks & Kemp, 2007; Diel & Lewis, 2022; O’Donnell & Bruce, 2001; Sandford & Bindemann, 2020). Indeed, familiarity is often tested by pairing a known face with a visually similar foil (e.g. Pozo et al., 2023; Robertson et al., 2016). Therefore, successful synthesis of familiar faces represents a particularly difficult task but also one with virtually limitless applications.

Synthesising realistic/believable images of familiar faces may be more challenging than the generation of unfamiliar ones because we are widely acknowledged to be experts with the former (Young & Burton, 2018). This expertise stems from stable representations tuned to specific identities (Burton et al., 2016) that, as a result, support recognition in even poor viewing conditions (Burton et al., 1999). However, it remains unclear as to whether this same expertise aids or impedes judgments of authenticity. If familiarity improves sensitivity to the information that defines a person’s unique appearance, it might help observers in detecting synthetic versions of that individual. Conversely, if familiarity fosters tolerance for natural variation (Brédart & Devue, 2006; Ge et al., 2003) then AI-synthesised images that match the identity’s appearance sufficiently may be accepted as genuine. Therefore, examining how familiarity might influence the detection of synthetic images represents an important test for established mechanisms of face perception when applied to this newly created class of AI-generated stimuli.

The distinction between synthesising unfamiliar versus familiar faces could also have practical implications. While synthesising new (fictional) faces may add credibility to fake online profiles (e.g. Park & Nicolau, 2015; Xu, 2014), the production of synthetic familiar faces would, for example, allow creators to manipulate celebrity endorsements, which can play a role in both marketing and political domains (see Knoll & Matthes, 2017). As a result, every image featuring a recognisable identity would require the viewer to question its authenticity. Such images fall within the broader category of ‘deepfakes’ (for a review, see Masood et al., 2023), which are becoming increasingly difficult to detect (Groh et al., 2022) and therefore pose a substantial challenge as technologies continue to advance.

OpenAI’s ChatGPT is a virtual assistant that has been adopted globally (around 250 million weekly active users; Jamali, 2024). While its capabilities are multifaceted, one recent addition allows for the generation of images from text. A description of the desired image (prompt) is entered into ChatGPT by the user and, behind the scenes, the chatbot processes this description and passes it to DALL·E (a diffusion model) for image generation. By training DALL·E on vast amounts of image–text pairs from the internet, the model learns patterns in these data (e.g. what makes a dog look like a dog) and is then able to generate new images given any description. Although the details of these training data have not been publicly disclosed, face images will likely have been well represented and so there is potential for synthetic face generation. Further, many online face images depict famous identities, meaning that DALL·E may have incorporated sufficient identity-specific information to allow for the generation of realistic images portraying these individuals.

To an extent, the realism of a generated image is limited only by the user’s ability to ‘engineer’ a high-quality prompt. Since people are relatively poor when tasked with providing detailed face descriptions (e.g. Kramer & Gous, 2020), ChatGPT’s vision model (GPT-4 V) can be exploited to assist in the process. An initial face image can be uploaded as input, which is then analysed by ChatGPT and provides a starting point for the generation of new images (e.g. by allowing the user to then refer to the original image’s style, background, or even facial appearance). Recent research has shown that GPT-4 V can perceive identity, emotion, and social traits from face images (e.g. Elyoseph et al., 2024; Kramer, 2025a, 2025b, 2025c, 2025d), and so this approach may facilitate the production of realistic face images.

### The current research

Here, we aimed to provide the first investigation of ChatGPT’s ability to generate realistic face images. Since recent work has shown that specialist algorithms like StyleGAN2 (Karras et al., 2021) are already capable of producing novel, fictional faces (e.g. Nightingale & Farid, 2022), we initially tasked ChatGPT (plus DALL·E) with this same goal. Crucially, GANs can only generate synthetic faces in the style of those images it was trained on, with the user typically unable to specify the desired characteristics of the output (e.g. the gender or ethnicity of the face, the background, etc.). In contrast, ChatGPT allows for complete control over image and face specifications, meaning that we could match synthetic images to real photographs and account for image properties that might influence judgements of realism (e.g. facial expression or background colour).

Moving beyond current demonstrations of novel face synthesis, we also aimed to explore the generation of familiar (celebrity) face images. As discussed earlier, this represents a significant paradigm shift in terms of potential applications but also brings with it substantial requirements. Synthetic images need to be believable both as face photographs *and* as instances of specific faces with which viewers have prior knowledge. If such images were indistinguishable from real photographs, this would bring into question the veracity of all online content moving forward while highlighting the need for society to develop solutions as a matter of urgency.

## Experiment 1

Previous studies using GANs have shown that images of fictional identities can be generated that are indistinguishable from real photos (e.g. Nightingale & Farid, 2022). However, synthesising faces in this way did not allow for image characteristics to be closely matched across synthetic and real faces. Here, we investigated a new method for generating synthetic images (i.e. using ChatGPT) that facilitated this matching. Again, we sought to determine whether our synthetic faces could be detected by viewers.

### Method

#### Participants

The sample sizes for our experiments were initially set as a compromise between available resources (i.e. participant payment) and our estimates of what was required to measure sufficiently precise effects with these experimental paradigms. In addition, we aimed to recruit samples comparable in size with previous work using a similar experimental design (see Experiment 1 of Miller et al., 2023).

Since we planned to use Bayesian methods for our central analyses, we anticipated increasing the sample size where estimates of theoretically important predictors were imprecise/ambiguous. It is worth noting that Bayesian methods do not suffer from many of the issues affecting frequentist analyses regarding optional stopping if the aim is simply sufficient precision rather than hypothesis confirmation (Rouder, 2014). In the end, our initial samples provided sufficiently unambiguous evidence and we did not choose to collect additional data.

We have also chosen to report summary participant performance (e.g. proportion correct, sensitivity, response bias) to allow readers to make broad comparisons with previous work in this field. To compare these values to a constant (e.g. chance performance), a one-sample *t*-test (two-tailed, α = 0.05, power = 95%) requires at least 54 participants to detect medium-sized effects (G*Power 3.1 software; Faul et al., 2007). In all experiments, our sample sizes exceeded this minimum requirement.

For this experiment, a sample of 110 participants (58 women, 51 men, 1 preferred another term; age *M* = 40.9 years, *SD* = 12.9 years; 76% self-reported ethnicity as White) were recruited through the Prolific online platform, where eligibility was restricted to those with an approval rate of 95% or above on the site. In addition, participation was limited to residents of the U.S., Canada, the U.K., Australia, or New Zealand. Participants’ data were excluded if they did not complete all trials; used a mobile phone (to avoid images appearing very small); responded incorrectly to at least one of the attention check trials; or gave the same response to all experimental trials (see details in the Supplemental Methods and Table S1 in the online supplementary materials). All participants in this research gave informed, onscreen consent before taking part and were provided with an onscreen debriefing upon completion. All experiments received ethical approval from the University of Lincoln’s research ethics committee (ref. 21014) and were carried out in accordance with the provisions of the World Medical Association Declaration of Helsinki. There was no overlap between participant samples across our four experiments.

#### Stimuli

Our real face photographs comprised a subset of the images used in previous research (Miller et al., 2023; Nightingale & Farid, 2022), originally taken from the Flickr-Faces-HQ Dataset (Karras et al., 2021). Nightingale and Farid (2022) divided their real images into 50 subsets, with each of these comprising eight images (with men and women, as well as Black, White, East Asian, and South Asian ethnicities, equally represented). We randomly selected 12 of these subsets (totalling 96 images) for use as our real photos.

To generate ‘matched’ synthetic images for these real photographs, we provided ChatGPT (model GPT-4o) with each photo, along with a crafted prompt. In brief, this asked ChatGPT to generate a new, fictional person while replicating both image (e.g. background, lighting) and person characteristics (e.g. gender, ethnicity). For the full prompt, see the Supplemental Methods in the online supplementary materials. Note that ChatGPT refused to produce images depicting children (who appeared in a small number of the real photos) and so we specified adult faces for our new images. Importantly, ChatGPT did not sample or reuse any part of the real photographs it was shown. In general terms, each photo served as a visual prompt (much like a detailed text prompt), which was then analysed and used to guide the generation of an entirely new image.

**Fig. 1:**
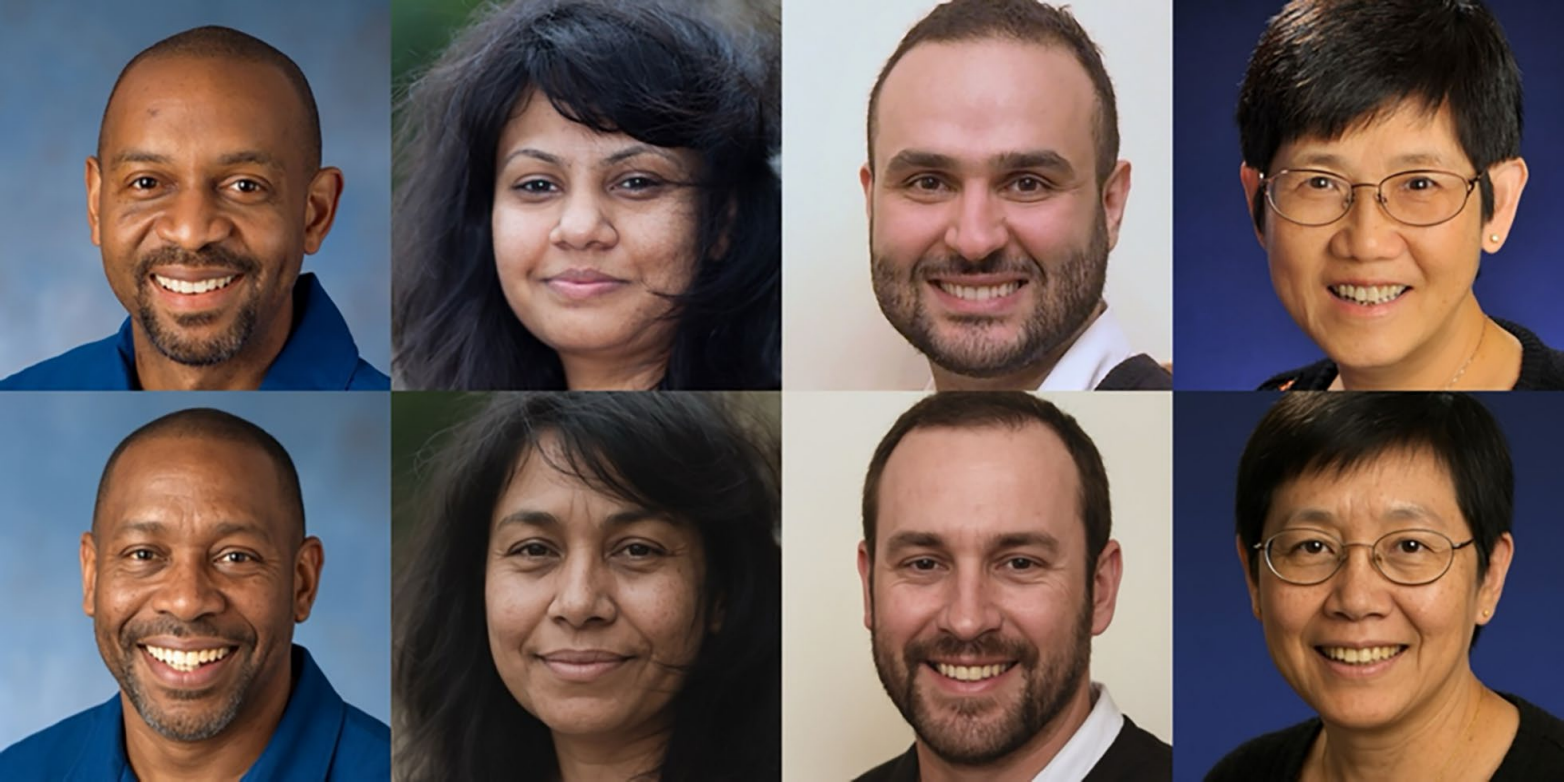
Example stimuli depicting matched image pairs from Experiment 1. Images are real (top row) and synthetic (bottom row). Real images were taken from the Flickr-Faces-HQ Dataset (Karras et al., 2021) and made available online by Nightingale and Farid (2022)

Synthetic images generated by ChatGPT were high quality, while the set of real photos showed some variation (e.g. in terms of lighting, blur, etc.). As such, our final step was to manually alter each synthetic image so that it more closely approximated its matched real photograph. The aim was to broadly equate the two matched images to rule out extraneous image characteristics when comparing how they were perceived, and that these adjustments would not have otherwise been required had the goal been to simply generate realistic images. (For example stimuli pre- vs. post-adjustment, see Fig. S1 in the online supplementary materials.) Using GIMP image editor (www.gimp.org), only general image settings (temperature, saturation, brightness, contrast, blur) were adjusted. Crucially, no changes were made to the synthetic images beyond these overall adjustments, and no changes were made to the real photographs. See Fig. 1 for example matched image pairs. Finally, all images were resized to 500 × 500 pixels, resulting in an experimental viewing size of 8.5 × 8.5 cm on a 24″ (1920 × 1080 pixel) display, for example.

To avoid individual participants viewing both images in a matched pair, our stimuli were divided into two sets. First, the 12 real face subsets (described above) were randomly split into two sets of six (while maintaining the eight image subsets themselves). Each matched synthetic image was then allocated to the opposite set to its real counterpart. In other words, Set A contained real photos of IDs 1–48 and the synthetic matched images for IDs 49–96, while Set B contained the inverse (synthetic for 1–48, real for 49–96).

#### Procedure

The experiment was completed using the Gorilla online testing platform (Anwyl-Irvine et al., 2020). After consent was obtained, participants provided demographic information (age, gender, ethnicity), as well as the type of device they were using. Next, participants were informed that they would be shown around 100 face images, and that they would be asked to decide whether each image was a real photograph or a completely new image generated by a computer. They were also told that a few obviously computer-generated images had been included to check they were paying attention.

During the task, each image was presented on screen individually below the question “is this a real photo or a computer-generated image?”, with these remaining until a response was given. The two response buttons, appearing below the image, were labelled ‘real photo’ and ‘computer-generated’. Responses were self-paced, and no feedback was given. Assignment to viewing either Set A (96 images) or Set B (96 images) was counterbalanced across participants, while the viewing order of the images was randomised for each participant. In addition, four attention check trials were included, where obvious distortions were present (see the Supplemental Methods and Fig. S2 in the online supplementary materials), and these were incorporated into the randomised order of each viewing sequence. Participants were also presented with a ‘halfway point’ screen after completing the first half of the task, informing them of their progress and providing an optional break before continuing.

#### Analytic strategy

We first investigated accuracy (proportion correct) on the task, along with signal detection measures (sensitivity and response bias). Since Nightingale and Farid (2022) provided image-level performance for the real face photos used here, we also considered whether our participants’ accuracies were associated with theirs.

Next, to more fully interpret the data, we applied model-based Bayesian inference to the disaggregated trial-level data using a hierarchical logistic regression model. Accuracy on each trial (1 = correct, 0 = incorrect) was predicted from two trial type fixed effects (synthetic vs real), plus a fixed effect of experiment condition (whether the participant viewed Set A or Set B) as a covariate. We also included a group-specific (random effect) structure to capture sources of variability across participants and identities, estimating the offset each participant and identity had in both the synthetic and real trial types.

The model was estimated with weakly informative priors on all model parameters (Gelman et al., 2017). A Bernoulli likelihood was used, allowing us to estimate the probability of a trial being correct, and is suitable given the binary outcome we observed. For the model coefficients (both trial types and condition), we used a Gaussian distribution with a mean of 0 and a standard deviation of 5. An LKJ Cholesky (Lewandowski et al., 2009) prior was used for the covariance matrix of the identity and participant group-specific effects – as such, we were also able to estimate the correlation between these effects, capturing, for example, whether an identity with particularly poor accuracy in the synthetic trials had similarly low accuracy in the real trials. This prior had an eta parameter equal to 2, with a half-Gaussian distribution with a standard deviation of 3 for the standard deviations of the group-specific effects. The model was estimated using PyMC (Abril-Pla et al., 2023) in the Python programming language. Four Markov Monte Carlo chains were run, with 1,000 tuning steps and 4,000 samples drawn from the posterior. Our model structure was:

$${\text{logit}}\left(\text{Pr}\left({Y}_{ijk}=1\right)\right)={\upbeta }_{ij}^{\text{Synthetic}}\cdot {x}_{i}^{\text{Synthetic}}+{\upbeta }_{ij}^{\text{Real}}\cdot {x}_{i}^{\text{Real}}+{\upbeta }_{\text{Condition}}\cdot {x}_{i}^{\text{Cond}}$$

where ${Y}_{ijk}$ is the accuracy on trial *k*, for identity *i* and participant *j*. The *x* variables are dummy-coded predictors, and the logit-transform converts the log-odds linear combination into probabilities.

To make inferences about our various hypotheses, we used the posterior probability of effects being in specific directions, calculated simply as the proportion of an effect being above or below zero, given the observed data and the model (Makowski et al., 2019). This was similar in intention to classical null-hypothesis significance testing but provided the probability that the hypothesis was different from zero given the data, and not the converse (Welsch et al., 2020). As logistic models have coefficients on the log-odds scale, we converted estimates to odds or probabilities to give clearer interpretation. We also estimated 94% highest-density intervals (HDIs) of all posterior estimates, which showed the credible range of effects given the observed data and model.

## Results

**Fig. 2:**
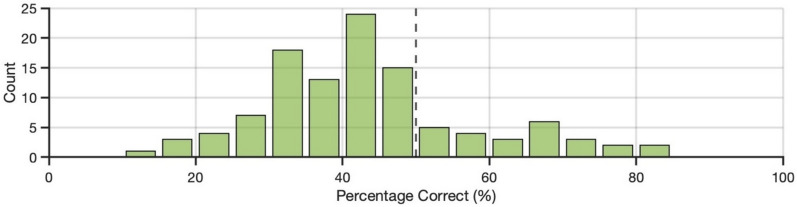
Variability in task accuracy for Experiment 1

Across all participants, accuracy (proportion correct) on the task (*M* = 0.43) was below-chance performance (of 0.50), *t*(109) = 4.76, *p* < 0.001, *d* = 0.45, 95% CI [0.26, 0.65] (see Fig. 2). Sensitivity (*d’*; *M* = − 0.50) was also below-chance performance (of 0), *t*(109) = 5.35, *p* < 0.001, *d* = 0.51, 95% CI [0.31, 0.71]. Finally, we found a positive response bias (criterion, *c*; *M* = 0.51), *t*(109) = 8.96, *p* < 0.001, *d* = 0.85, 95% CI [0.63, 1.07], indicating that participants were biased to respond with ‘real photo’ during the task.

Since Nightingale and Farid (2022) provided image-level performance for the real face photos used here, we considered whether our participants’ accuracies were associated with theirs. To this end, we calculated the proportion correct for each of the 96 real photos using their dataset (available online) and, separately, our own. Across these images, we found a strong association between performances derived from the two participant samples, *r*(94) = 0.66, 95% CI = [0.53, 0.76], *p* < 0.001. Further, a comparison of the two sets of image-level accuracies showed that our sample (*M* = 0.58) produced significantly higher values than theirs (*M* = 0.51), *t*(95) = 4.96, *p* < 0.001, *d* = 0.51, 95% CI = [0.29, 0.72]. Taken together, these results provide evidence that our participants, while performing poorly on the task, were not simply responding at random.

### Can observers distinguish between real and synthetic images?

Marginalising over all predictors (trial type and condition), the baseline performance on the task was *M* = 0.41, 94% CrI [0.37, 0.44], *p*(H < 0.5) = 100%, i.e. below chance (0.5). Next, we inspected the posterior estimates of the trial type coefficients from the model, representing the average baseline accuracy in each trial type, and considered the posterior probability that these accuracies were above or below chance. The model-estimated probability of identifying a real image as real was *M* = 0.62, 94% CrI [0.55, 0.68], *p*(H > 0.5) = 99.9%, and for identifying synthetic as synthetic, *M* = 0.20, 94% CrI [0.15, 0.25], *p*(H < 0.5) = 100%. As such, across participants and identities, accuracy for real images was above chance, while for synthetic images, it was clearly below. That participants mistook these AI-generated novel faces for real photos indicated they were clearly plausible.

## Experiment 2

Previous studies found that computer-generated face images were indistinguishable from real photographs (e.g. Miller et al., 2023; Nightingale & Farid, 2022). However, their method of generation (i.e. using StyleGAN2) did not allow for the specification of image or face characteristics. In our first experiment, we introduced a new way to generate face images (i.e. using ChatGPT) that provided this level of control while successfully producing images that participants could not detect as computer-generated.

In Experiment 2, we used this same approach to image generation but took an additional step. For the first time, we investigated the potential for synthesising images of familiar (famous) faces. In other words, can ChatGPT be used to generate images that are believable both as face photographs *and* as instances of specific faces with which viewers have prior knowledge? If the chatbot demonstrates this capability then such a result has far-reaching implications.

### Method

#### Participants

A sample of 115 participants (59 women, 55 men, 1 preferred another term; age *M* = 44.4 years, *SD* = 13.8 years; 71% self-reported ethnicity as White) were recruited online. All eligibility and exclusion criteria were identical to Experiment 1. (For details of exclusions, see the Supplemental Methods and Table S1 in the online supplementary materials.)

#### Stimuli

We collected photographs of 100 (50 men, 50 women; varied ethnicities) internationally famous celebrities (predominantly Hollywood actors). For each identity, we downloaded a large, high-quality photograph using Google Images searches, with each image depicting the individual facing roughly front-on and with their face free from occlusions. To generate ‘matched’ synthetic images for these real photographs, we followed the same process as in Experiment 1. However, the prompt we used here asked ChatGPT to generate a new image of the person depicted in the original photograph, changing the pose but replicating all other details about the image (e.g. the background). For the full prompt, see the Supplemental Methods in the online supplementary materials. Note that ChatGPT refused to generate images of named celebrities when asked, and so we did not identify these individuals during the process. Even so, it was clear that ChatGPT recognised the identities in that, on occasion, it would refuse to generate a new image of specific individuals (e.g. Tom Hanks), in which case we chose other identities as replacements. As in Experiment 1, each photo served as a visual prompt, which was then analysed and used to guide the generation of an entirely new image based on the original one.

**Fig. 3:**
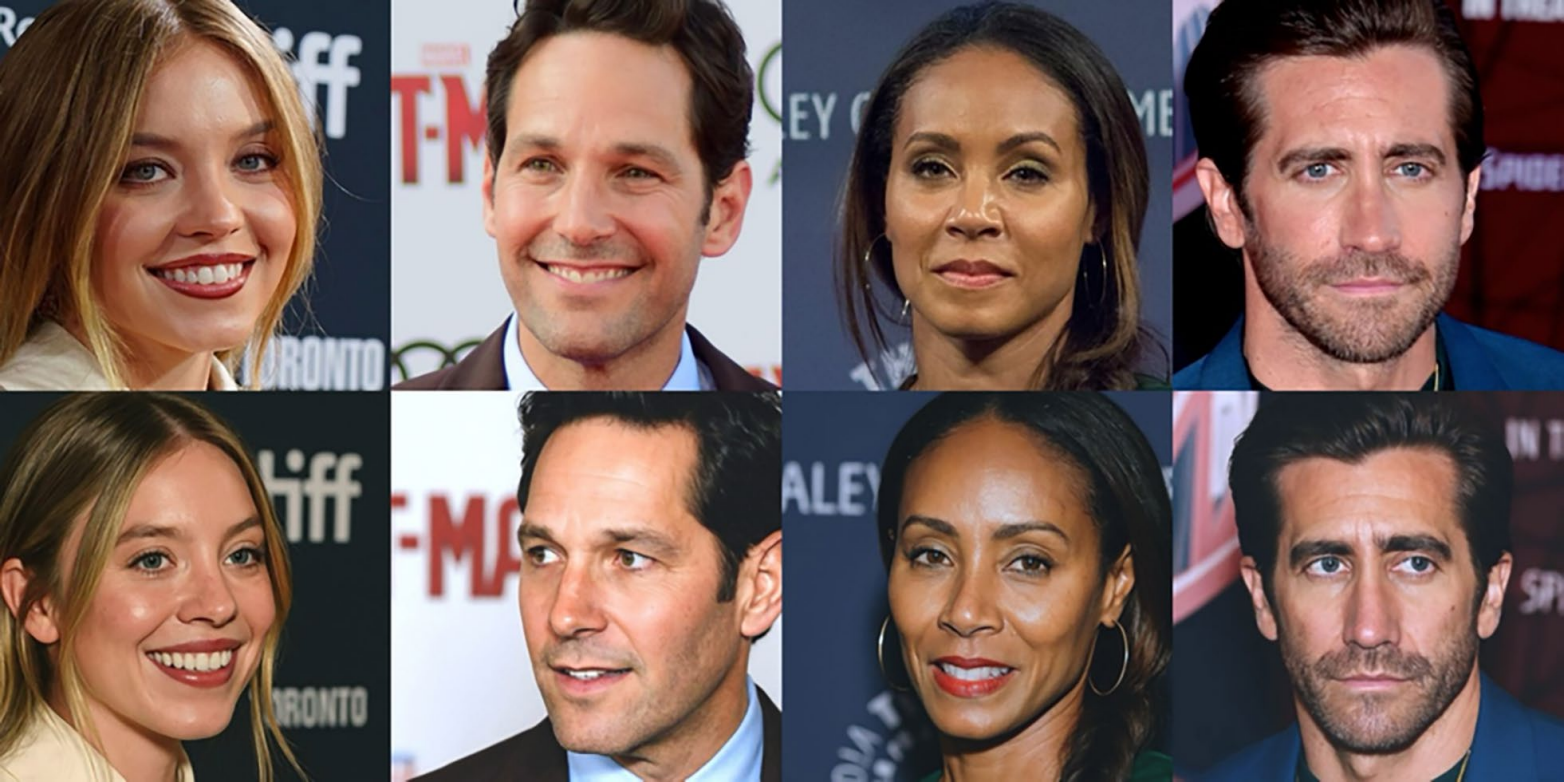
Example stimuli depicting matched image pairs from Experiments 2–4*.* Images are real (top row) and synthetic (bottom row). Image attributions (top row left to right): Jay Dixit (cropped); Red Carpet Report on Mingle Media TV (cropped); Dominick D (cropped); Toglenn (cropped). Photographs are from Wikimedia Commons (2025) (https://commons.wikimedia.org/)

Again, we manually altered the general image properties for each synthetic image so that it more closely approximated its matched real photograph. Next, each matched pair of images was similarly cropped to contain only the head and neck, and in some cases, the top of the shoulders. (For example stimuli pre- vs. post-adjustment, see Fig. S1 in the online supplementary materials.) See Fig. 3 for example matched image pairs. Finally, all images were resized to 500 × 500 pixels, with the same experimental viewing size as in Experiment 1.

To avoid individual participants viewing both images in a matched pair, our stimuli were again divided into two sets. First, the 100 real face photographs were randomly split into two sets of 50 with the proviso that each set contained an equal number of men and women. Each matched synthetic image was then allocated to the opposite set to its real counterpart. In other words, Set A contained real photos of IDs 1–50 and the synthetic matched images for IDs 51–100, while Set B contained the inverse (synthetic for 1–50, real for 51–100).

#### Procedure

The general procedure was identical to Experiment 1, with the following caveats. First, each trial began with a fixation cross, displayed for 500 ms. This served to provide participants with a clear separation between trials since these now involved two judgements (decision + rating). Second, the onscreen instruction appearing above each image incorporated the name of the identity depicted. For example, “is this a real photo or a computer-generated image of Paul Rudd?” Third, following the participant’s binary response (‘real photo’ or ‘computer-generated’), the image was removed and a new instruction asked “before this experiment, how familiar were you with the facial appearance of Paul Rudd?” (or whichever identity appeared in the image on the previous screen). Participants responded using a 7-point scale with labelled anchors (1 = extremely unfamiliar to 7 = extremely familiar).

As in Experiment 1, assignment to viewing either Set A (100 images) or Set B (100 images) was counterbalanced across participants, while the viewing order of the images was randomised for each participant. Again, four attention check trials were included, where obvious distortions were present (see the Supplemental Methods and Fig. S3 in the online supplementary materials), and these were incorporated into the randomised order of each viewing sequence.

#### Analytic strategy

As in Experiment 1, we first investigated accuracy (proportion correct) on the task, along with signal detection measures (sensitivity and response bias). Next, and also mirroring Experiment 1, we relied on model-based Bayesian inference to interpret the data, again with a hierarchical logistic regression model, predicting accuracy on each trial. The model here was expanded from the two trial type fixed effects (synthetic vs real) and condition covariate to include a familiarity predictor (in natural rating scale units, i.e. 1, 2 … 7) and its interaction with trial type. This allowed us to investigate the influence of familiarity on accuracy for both real and synthetic images. The group-specific (random effect) structure was identical to Experiment 1, including offsets for participant and identities around the two trial types.

The prior distribution and sampling settings were identical to Experiment 1. Our model structure was:

$${\text{logit}}\left(\text{Pr}\left({Y}_{ijk}=1\right)\right)={\beta }_{ij}^{\text{Synthetic}}\cdot {x}_{i}^{\text{Synthetic}}+{\beta }_{ij}^{\text{Real}}\cdot {x}_{i}^{\text{Real}}+{\gamma }^{\text{Synthetic}}\cdot {{\text{Fam}}{\text{iliarity}}}_{i}^{\text{Synthetic}}+{\gamma }^{\text{Real}}\cdot {\text{Familiarity}}_{i}^{\text{Real}}+{\upbeta }_{\text{Condition}}\cdot {x}_{i}^{\text{Cond}}$$

where ${Y}_{ijk}$ is the accuracy on trial *k*, for identity *i* and participant *j*. The *x* variables are dummy-coded predictors representing trial types, and the $\gamma$ variables are familiarity ratings under each of the trial types, allowing the model to directly estimate the influence of familiarity on both trial types separately.

## Results

**Fig. 4:**
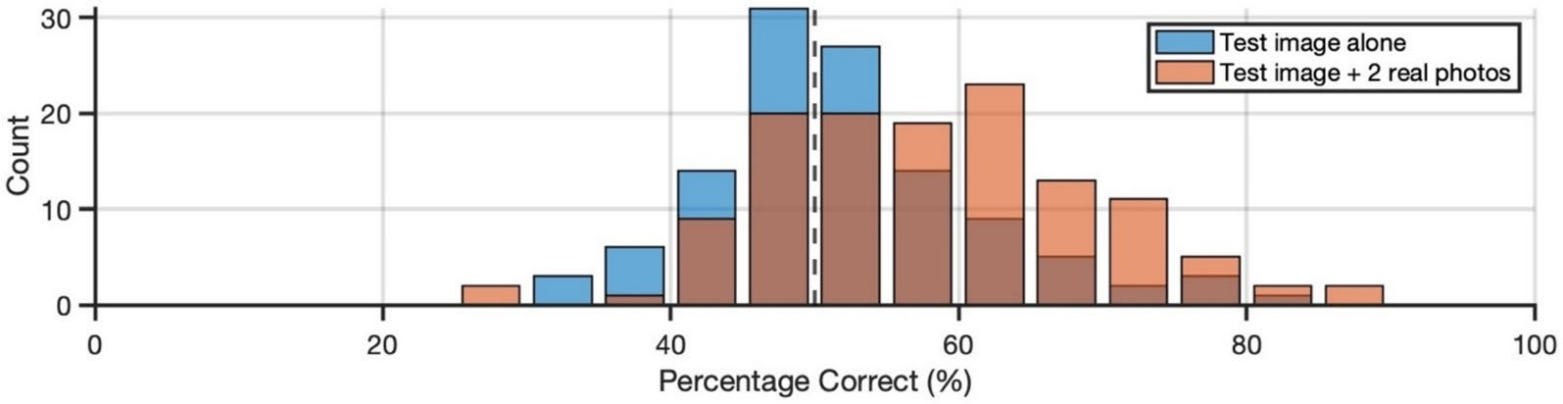
Variability in task accuracy for Experiments 2 and 3*.* Performance is displayed for Experiment 2 (blue) and Experiment 3 (orange)

First, we calculated simple performance (ignoring possible familiarity effects). Across all participants, accuracy (proportion correct) on the task (*M* = 0.52) was no different from chance performance (of 0.50), *t*(114) = 1.74, *p* = 0.084, *d* = 0.16, 95% CI [− 0.02, 0.35] (see Fig. 4). Sensitivity (*d’*; *M* = 0.10) was also no different from chance performance (of 0), *t*(114) = 1.67, *p* = 0.098, *d* = 0.16, 95% CI [− 0.03, 0.34]. Finally, as with Experiment 1, we found a positive response bias (criterion, *c*; *M* = 0.49), *t*(114) = 7.25, *p* < 0.001, *d* = 0.68, 95% CI [0.47, 0.88], indicating that participants were biased to respond with ‘real photo’ during the task.

### Can observers distinguish between real and synthetic images?

Marginalising over all predictors, the average accuracy on the task was *M* = 0.51, 94% CrI [0.48, 0.54] *p*(H > 0.5) = 67%, i.e. at the level of chance. Next, we inspected the posterior estimates of the trial type coefficients, which represented baseline accuracy in the model, and calculated the probability that these were above or below chance. The model-estimated probability of identifying a real image as real was *M* = 0.70, 94% CrI [0.64, 0.75], *p*(H > 0.5) = 100%, and for identifying synthetic as synthetic, *M* = 0.32, 94% CrI [0.26, 0.38], *p*(H < 0.5) = 100%.

### Does familiarity improve accuracy?

**Fig. 5:**
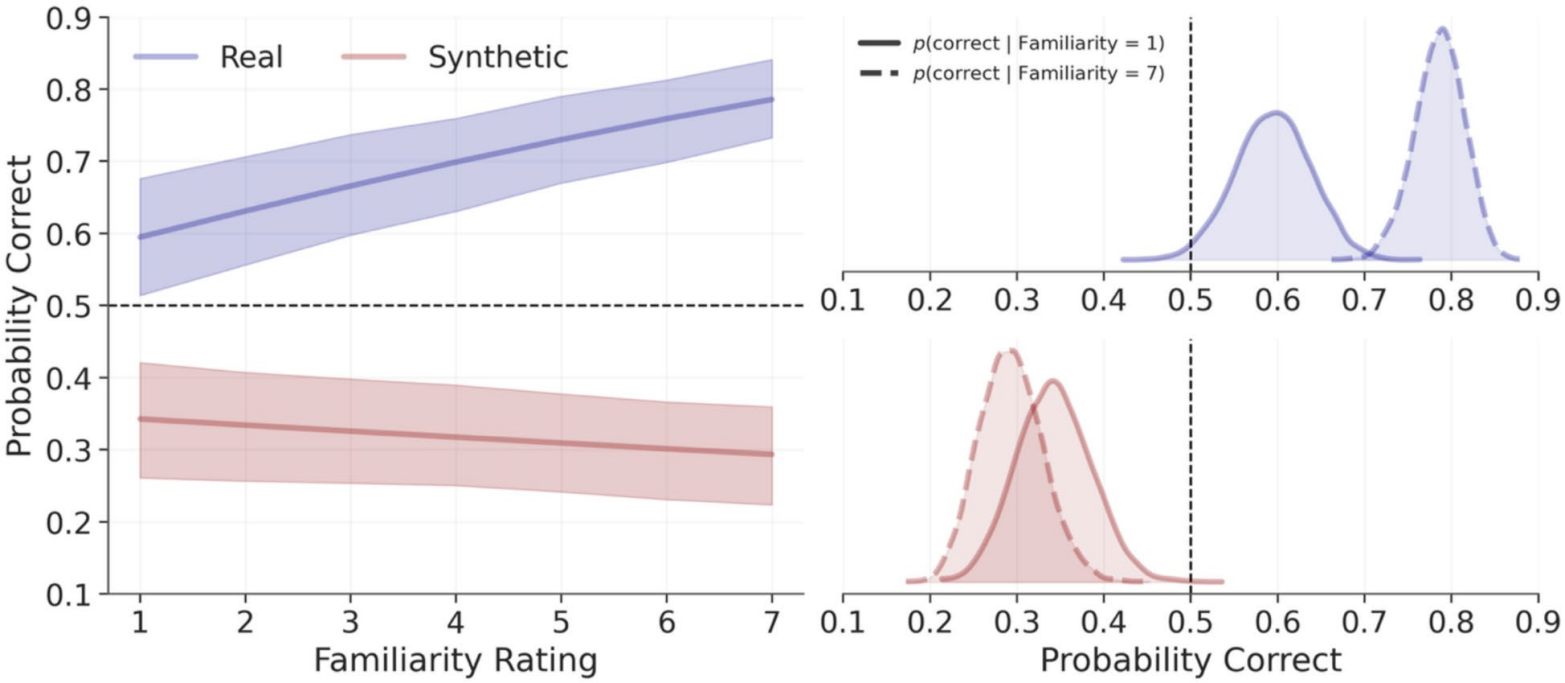
Model predictions for the influence of familiarity on accuracy in Experiment 2. The left panel depicts model predictions, where shaded areas represent 94% credible intervals, with synthetic trials shown in red and real trials shown in blue. The right panel depicts posterior distributions of conditional model predictions, setting familiarity to the lowest (one) and highest (seven) levels. The dashed vertical lines represent accuracies expected by chance

To investigate whether familiarity improved accuracy, we converted the model-estimated slopes to odds (see Fig. 5). For real trials, a one-point increase in familiarity was associated with a 21% increase in the likelihood of a correct response, *OR* = 1.21, 94% CrI [1.14, 1.28], *p*(H > 1) = 100%, while for synthetic trials, increasing familiarity was associated with a 4% reduction in the likelihood of a correct response, *OR* = 0.96, 94% CrI [0.93, 1], *p*(H < 1) = 95.8%. (For a visualisation of the raw data prior to modelling, see the Supplemental Results and Fig. S4 in the online supplementary materials.)

However, given that logistic models are nonlinear, interpreting coefficients directly should be done with caution, as changes across variables are not constant. To aid interpretation, for each trial type, we used the model to predict accuracy when fixing familiarity to one (lowest possible familiarity) and seven (highest possible). For real trials, low familiarity had clearly above-chance accuracy, *M* = 0.59, 94% CrI [0.51, 0.68], *p*(H > 0.5) = 98.5%, as did high familiarity, *M* = 0.79, 94% CrI [0.73, 0.84], *p*(H > 0.5) = 100%. For synthetic trials, low familiarity had clearly below-chance accuracy, *M* = 0.34, 94% CrI [0.26, 0.42], *p*(H < 0.5) = 99.9%, as did high familiarity, *M* = 0.29, 94% CrI [0.22, 0.36], *p*(H < 0.5) = 100%. From these estimates we computed the relative increase afforded by familiarity by dividing the maximum by the minimum (i.e. the relative risk). For real trials, a correct response was 1.33 times, 94% CrI [1.19, 1.45], *p*(H > 1) = 100%, as likely to occur under high versus low familiarity. For synthetic trials, a correct response was 0.86 times, 94% CrI [0.73, 1] *p*(H < 1) = 95.8%, as likely. As such, familiarity benefited performance with real photos but was detrimental for synthetic images (see Fig. 5).

## Experiment 3

Prior familiarity with identities provided only limited benefits when tasked with differentiating between real photos and computer-generated images. This may be due to participants having to rely on their internal representations of these identities, which are sufficiently robust as to support accurate identification (e.g. Kramer et al., 2018) but failed to substantially benefit performance here. Therefore, we next investigated whether displaying additional (real) photos of the identity alongside those seen in Experiment 2 would increase synthetic image detection. These additional photographs onscreen (1) gave viewers facial appearance information for comparison without the need to rely on memory, and (2) represented a likely real-world scenario since computer-generated images posted online will either be seen alongside real photos or, at the very least, available for comparison with them.

### Method

#### Participants

A sample of 127 participants (52 women, 75 men; age *M* = 40.1 years, *SD* = 12.4 years; 69% self-reported ethnicity as White) were recruited online. All eligibility and exclusion criteria were identical to Experiment 1. (For details of exclusions, see the Supplemental Methods and Table S1 in the online supplementary materials.)

#### Stimuli

We collected two additional photographs of each celebrity featured in Experiment 2, following the same procedure as in the original collection of images. These new images were also cropped and resized to 500 × 500 pixels, with the same experimental viewing size as those in Experiments 1 and 2.

#### Procedure

**Fig. 6:**
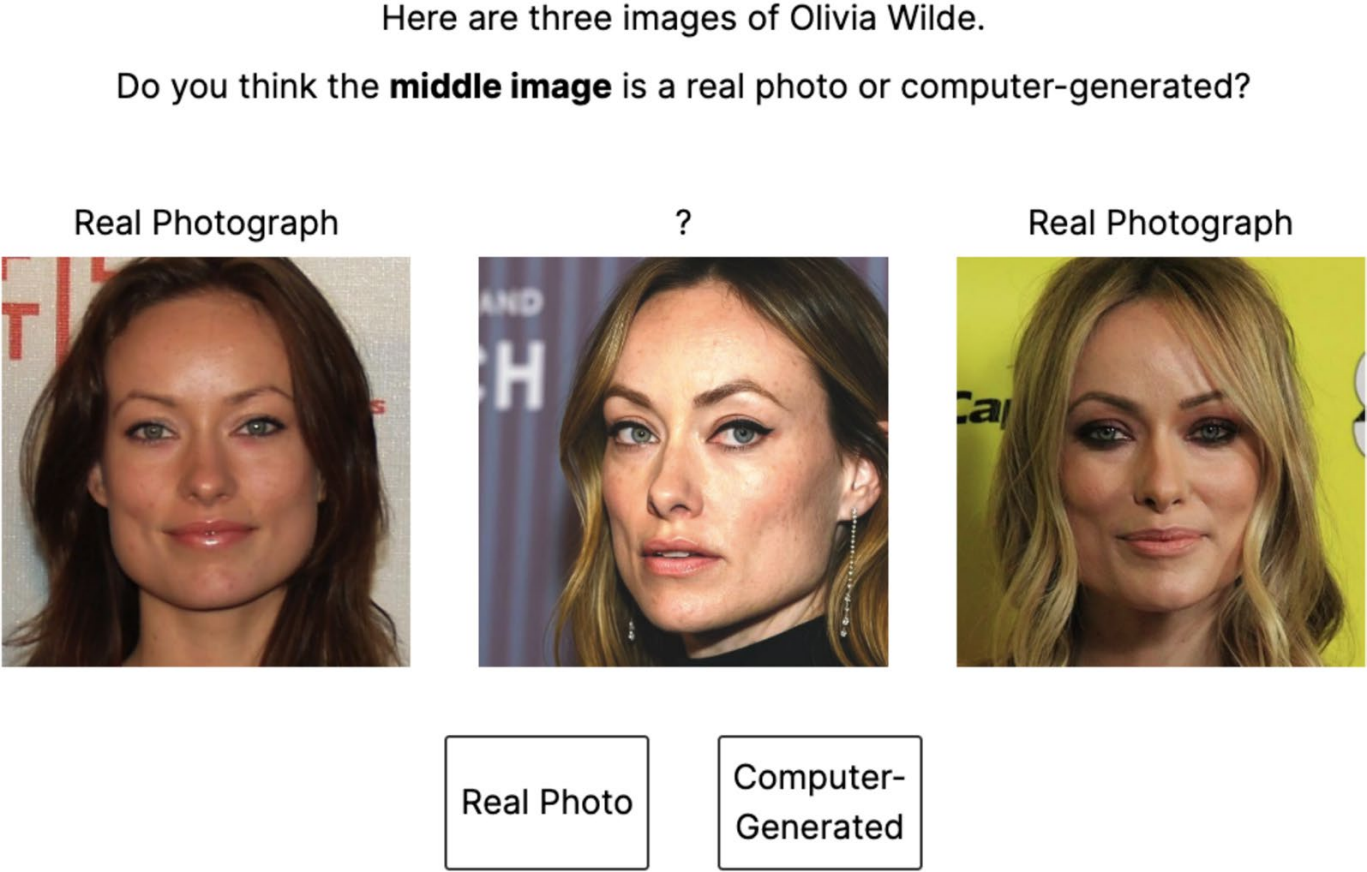
An example trial from Experiment 3*.* The correct answer here is ‘computer-generated’. Image attributions: left image—David Shankbone (cropped); right image—DannyB Photos (cropped). Photographs are from Wikimedia Commons (2025) (https://commons.wikimedia.org/)

The general procedure was identical to Experiment 2, except in this case, the two-alternative forced choice (real or computer-generated) was directed towards the middle of three images. As such, we incorporated the following changes. First, instructions before the task explained that on each trial, participants were to decide whether the middle image was a real photograph or a completely new image generated by a computer, and that two real photographs would also be provided to help with their decision-making. Second, during the task, three images for a given identity were presented onscreen next to each other with the instruction appearing above them asking, for example, “Here are three images of Paul Rudd. Do you think the *middle image* is a real photo or computer-generated?” Third, the two additional real images were presented on either side of the image in question, with the label “real photograph” shown above them (see Fig. 6). After making their response, participants provided a familiarity rating as in Experiment 2.

Assignment to viewing either Set A (100 identities) or Set B (100 identities) was counterbalanced across participants, while the viewing order of the identities was randomised for each participant. Finally, four attention check trials were included using those images featured in Experiment 2 (see the Supplemental Methods and Fig. S3 in the online supplementary materials), although here presented in the middle of two real photos of the identity. These were incorporated into the randomised order of each viewing sequence.

#### Analytic strategy

Again, we investigated accuracy (proportion correct) on the task, along with signal detection measures (sensitivity and response bias). For our model-based Bayesian inference, the model structure was identical to that of Experiment 2, including all group-specific effects, priors, and sampling approaches.

## Results

First, we calculated simple performance (ignoring possible familiarity effects). Across all participants, accuracy (proportion correct) on the task (*M* = 0.58) was significantly greater than chance performance (of 0.50), *t*(126) = 7.86, *p* < 0.001, *d* = 0.70, 95% CI [0.50, 0.89] (see Fig. 4). Sensitivity (*d’*; *M* = 0.46) was also greater than chance performance (of 0), *t*(126) = 7.66, *p* < 0.001, *d* = 0.68, 95% CI [0.49, 0.87]. Finally, we found a small, positive response bias (criterion, *c*; *M* = 0.10), *t*(126) = 2.16, *p* = 0.033, *d* = 0.19, 95% CI [0.02, 0.37], indicating that participants were somewhat biased to respond with ‘real photo’ during the task.

### Can observers distinguish between real and synthetic images?

Marginalising over all predictors, the average accuracy on the task was *M* = 0.59, 94% CrI [0.56, 0.62], *p*(H > 0.5) = 100%, i.e. above chance levels. Next, we inspected the posterior estimates of the trial type coefficients, which represented baseline accuracy in the model, and calculated the probability that these were above or below chance. The model-estimated probability of identifying a real image as real was *M* = 0.62, 94% CrI [0.58, 0.67], *p*(H > 0.5) = 100%, and for identifying synthetic as synthetic, *M* = 0.56, 94% CrI [0.50 0.61], *p*(H > 0.5) = 96.2%.

### Does familiarity improve accuracy?

**Fig. 7:**
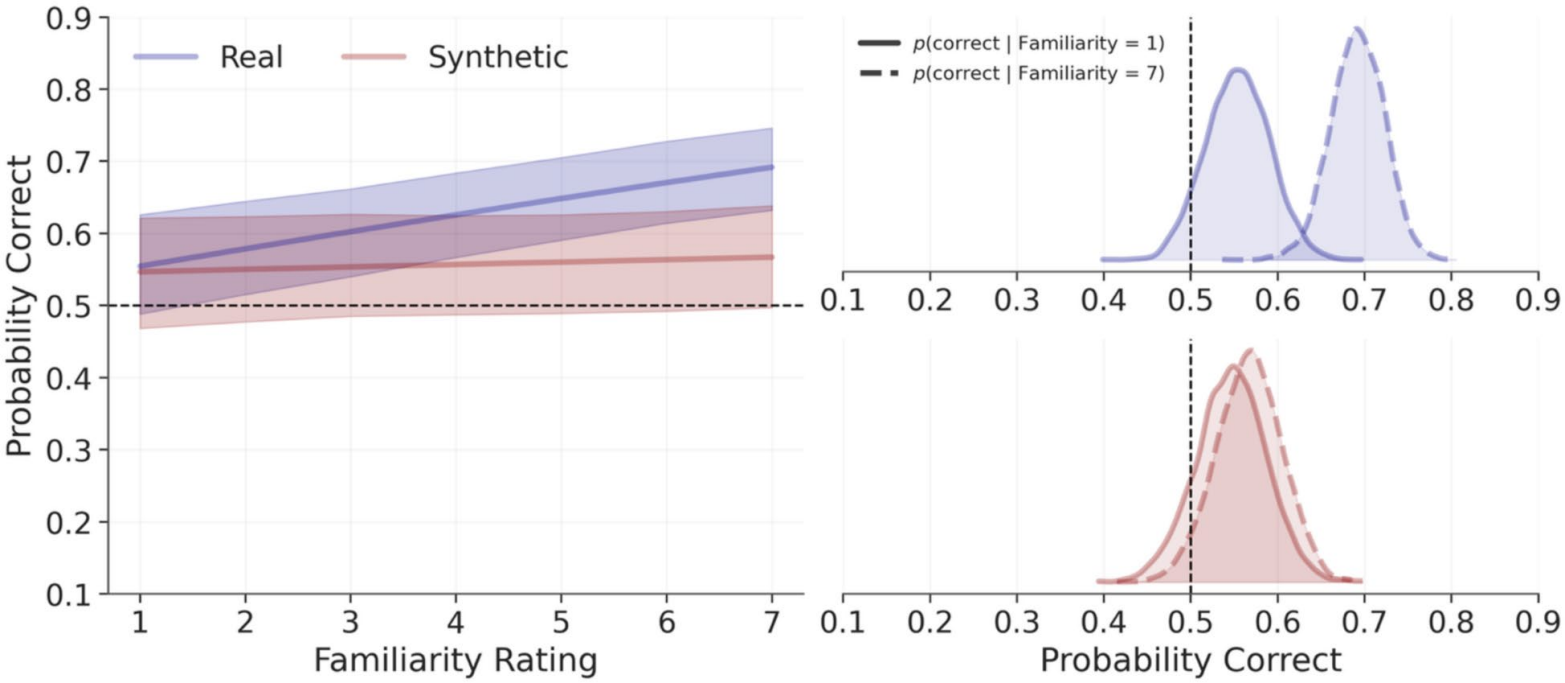
Model predictions for the influence of familiarity on accuracy in Experiment 3*.* The left panel depicts model predictions, where shaded areas represent 94% credible intervals, with synthetic trials shown in red and real trials shown in blue. The right panel depicts posterior distributions of conditional model predictions, setting familiarity to the lowest (one) and highest (seven) levels. The dashed vertical lines represent accuracies expected by chance

To aid interpretation, we again converted the model-estimated slopes to odds (see Fig. 7). For real trials, a one-point increase in familiarity was associated with a 9% increase in the likelihood of a correct response, *OR* = 1.09, 94% CrI [1.04, 1.15], *p*(H > 1) = 99.9%, while for synthetic trials, increasing familiarity was associated with a 1% increase in the likelihood of a correct response, *OR* = 1.01, 94% CrI [0.98, 1.05], *p*(H > 1) = 75.4%, though this was not definitely positive. (For a visualisation of the raw data prior to modelling, see the Supplemental Results and Fig. S5 in the online supplementary materials.)

Next, for each trial type, we used the model to predict accuracy when fixing familiarity to 1 (lowest possible familiarity) and 7 (highest possible). For real trials, low familiarity showed a point estimate above chance but with credible intervals overlapping chance, *M* = 0.55, 94% CrI [0.49, 0.63], *p*(H > 0.5) = 92.8%, while high familiarity was clearly above chance, *M* = 0.69, 94% CrI [0.63, 0.75], *p*(H > 0.5) = 100%. For synthetic trials, low familiarity was also above chance but again with credible intervals overlapping chance, *M* = 0.55, 94% CrI [0.47, 0.62], *p*(H > .5) = 87.2%, while high familiarity was somewhat higher and more certainly above chance, *M* = 0.57, 94% CrI [0.50, 0.64], *p*(H > .5) = 95.7% From these estimates we computed the relative increase afforded by familiarity by dividing the maximum by the minimum (i.e. the relative risk). For real trials, a correct response was 1.25 times, 94% CrI [1.14, 1.36], *p*(H > 1) = 100%, as likely to occur under high versus low familiarity. For synthetic trials, a correct response was essentially even, *M* = 1.04, 94% CrI [0.93, 1.15], *p*(H > 1) = 75.4%, though the posterior estimate suggests a higher probability of a correct response occurring more under high familiarity. Taken together, familiarity resulted in an overall improvement in accuracy (see Fig. 7).

### How does the addition of two real, reference photos influence performance?

Since the only difference between this experiment and Experiment 2 was the presence of two additional, real photographs on each trial, we were able to combine datasets from these two experiments in a single model. This model was identical to the one used above, except for two changes. First, we included an interaction with a categorical ‘experiment’ indicator, allowing the trial type baseline accuracy to vary across experiments, as well as the association between familiarity and trial type. Second, we expanded the group-specific random effects structure for identities, which now appeared under both trial types for each experiment.

Marginalising over all predictors, the model-predicted accuracy here was about 1.17 times, 94% CrI [1.10, 1.24], *p*(H > 1) = 100%, greater relative to Experiment 2. Next, we compared model predictions for each trial type under each experiment, marginalised over familiarity. For real trials, the predicted accuracy here showed evidence of a relative decrease by 0.90, 94% CrI [0.81 0.99], *p*(H < 1) = 97.3%, in comparison with Experiment 2. For synthetic trials, the presence of additional images here clearly increased accuracy by 1.76 times, 94% CrI [1.45, 2.11], *p*(H > 1) = 100%, in comparison with Experiment 2. Taken together, these findings demonstrated a definite increase in accuracy with the addition of two real photographs, resulting mostly from their beneficial effect on trials involving synthetic images.

## Experiment 4

Providing two additional, real photographs led to an increase in performance for synthetic image detection, presumably by allowing for a comparison with these images in addition to any internal representation previously developed through familiarity. However, in everyday contexts, additional images are unlikely to be accompanied by ‘real photograph’ labels and, as such, their authenticity may also be unknown. Therefore, in this final experiment, we investigated performance for the same three-image displays without providing such labels. Instead, and mirroring a lineup-style task, participants were unaware of whether one image was computer-generated (i.e. ‘target present’) or all three images were real photographs (i.e. ‘target absent’).

### Method

#### Participants

A sample of 120 participants (49 women, 70 men, 1 preferred another term; age *M* = 39.0 years, *SD* = 13.4 years; 68% self-reported ethnicity as White) were recruited online. All eligibility and exclusion criteria were identical to Experiment 1. (For details of exclusions, see the Supplemental Methods and Table S1 in the online supplementary materials.)

#### Stimuli

The stimuli were those featured in Experiment 3, providing us with a ‘target present’ and a ‘target absent’ lineup for each identity (where the synthetic image was the ‘target’). Both lineups for a given identity contained the two additional, real photographs, alongside either the original photo or the synthetic image from the matched pair.

To avoid individual participants viewing both lineups for a given identity, we continued to use the two sets of 50 identities created in Experiment 2. Here, Set A contained ‘target absent’ lineups for IDs 1–50 and ‘target present’ lineups for IDs 51–100, while Set B contained the inverse (‘present’ for 1–50, ‘absent’ for 51–100).

#### Procedure

The general procedure was identical to Experiment 3, except in this case, the task was a four-alternative forced choice (deciding whether one or none of the three images were computer-generated). As such, we incorporated the following changes. First, instructions before the task explained that on each trial, either one image was a completely new image generated by a computer or all of the images were real photographs (so none were computer generated). Second, during the task, the three lineup images for a given identity were presented onscreen next to each other with the instruction appearing above them asking, for example, “Here are three images of Paul Rudd. Do you think any of them are computer-generated?” Third, numbered labels appeared above the three images (from left to right): ‘Image 1’, ‘Image 2’, ‘Image 3’. Fourth, participants gave a four-alternative forced choice response after being presented with the following options: ‘image 1 is computer-generated’, ‘image 2 is computer-generated’, ‘image 3 is computer-generated’, ‘all images are real photos’. (Only one option could be selected.) Following this response, participants provided a familiarity rating as in Experiments 2 and 3.

Assignment to viewing either Set A (100 lineups) or Set B (100 lineups) was counterbalanced across participants, while the viewing order of the lineups was randomised for each participant. In addition, the location of the three lineup images onscreen (left/middle/right) was randomised for every trial. Finally, four attention check trials were included using those featured in Experiment 3 (see the Supplemental Methods and Fig. S3 in the online supplementary materials). These were incorporated into the randomised order of each viewing sequence.

#### Analytic strategy

Again, we investigated accuracy (proportion correct) on the task, along with signal detection measures (sensitivity and response bias). For our model-based Bayesian inference, the model structure was identical to that of Experiments 2 and 3, including all group-specific effects, priors, and sampling approaches.

## Results

**Fig. 8:**
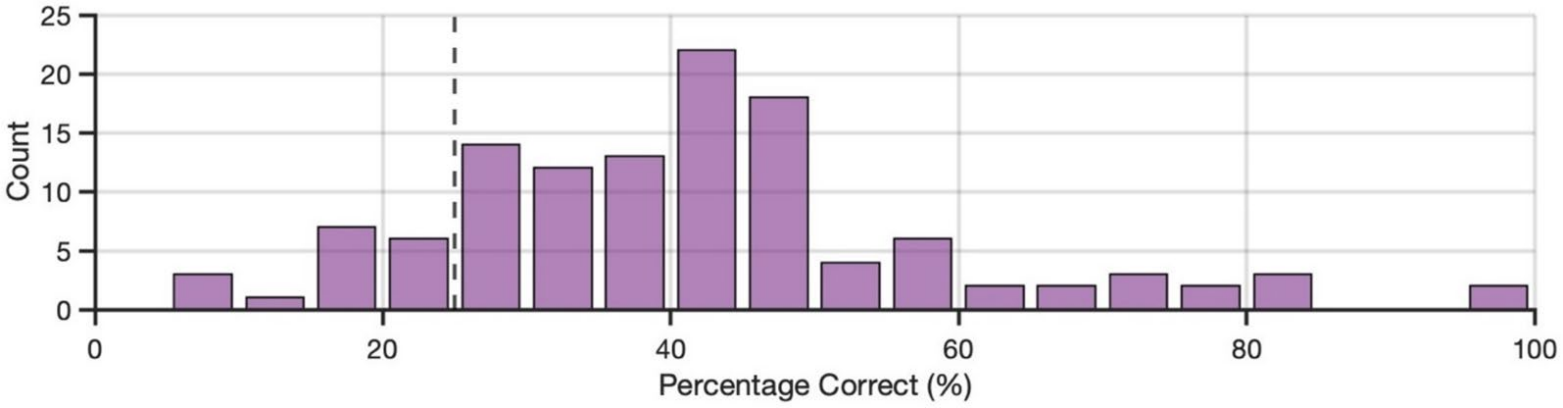
Variability in task accuracy for Experiment 4

First, we calculated simple performance (ignoring possible familiarity effects). Correct responses were as follows: 1) a synthetic image was present and participants identified it as synthetic (a ‘hit’), or 2) no synthetic image was present and participants responded with ‘all images are real photos’ (a ‘correct rejection’). Across all participants, accuracy (proportion correct) on the task (*M* = 0.41) was significantly greater than chance performance (of 0.25), *t*(119) = 10.38, *p* < 0.001, *d* = 0.95, 95% CI [0.73, 1.16] (see Fig. 8). Considering each trial type separately, both ‘target present’ trials (*M* = 0.33), *t*(119) = 3.47, *p* < 0.001, *d* = 0.32, 95% CI [0.13, 0.50], and ‘target absent’ trials (*M* = 0.50), *t*(119) = 10.90, *p* < 0.001, *d* = 1.00, 95% CI [0.78, 1.21], were also at above-chance performance. For information on the five response outcomes that were possible, see the Supplemental Results and Figure S6 in the online supplementary materials.

### How well do observers perform on the task?

Marginalising across all predictors, overall accuracy on the task was generally low but excluded chance performance, *M* = 0.38, 94% CrI [0.34, 0.43], *p*(H > 0.25) = 100%. Consideration of the posterior estimates of the trial type coefficients showed that for ‘target absent’ trials, the model-estimated probability of accuracy was clearly greater than chance, *M* = 0.49, 94% CrI [0.43, 0.56], *p*(H > 0.25) = 100%, but ‘target present’ trials were essentially at chance, *M* = 0.26, 94% CrI [0.21, 0.32], *p*(H > 0.25) = 68.9%.

### Does familiarity improve accuracy?

**Fig. 9:**
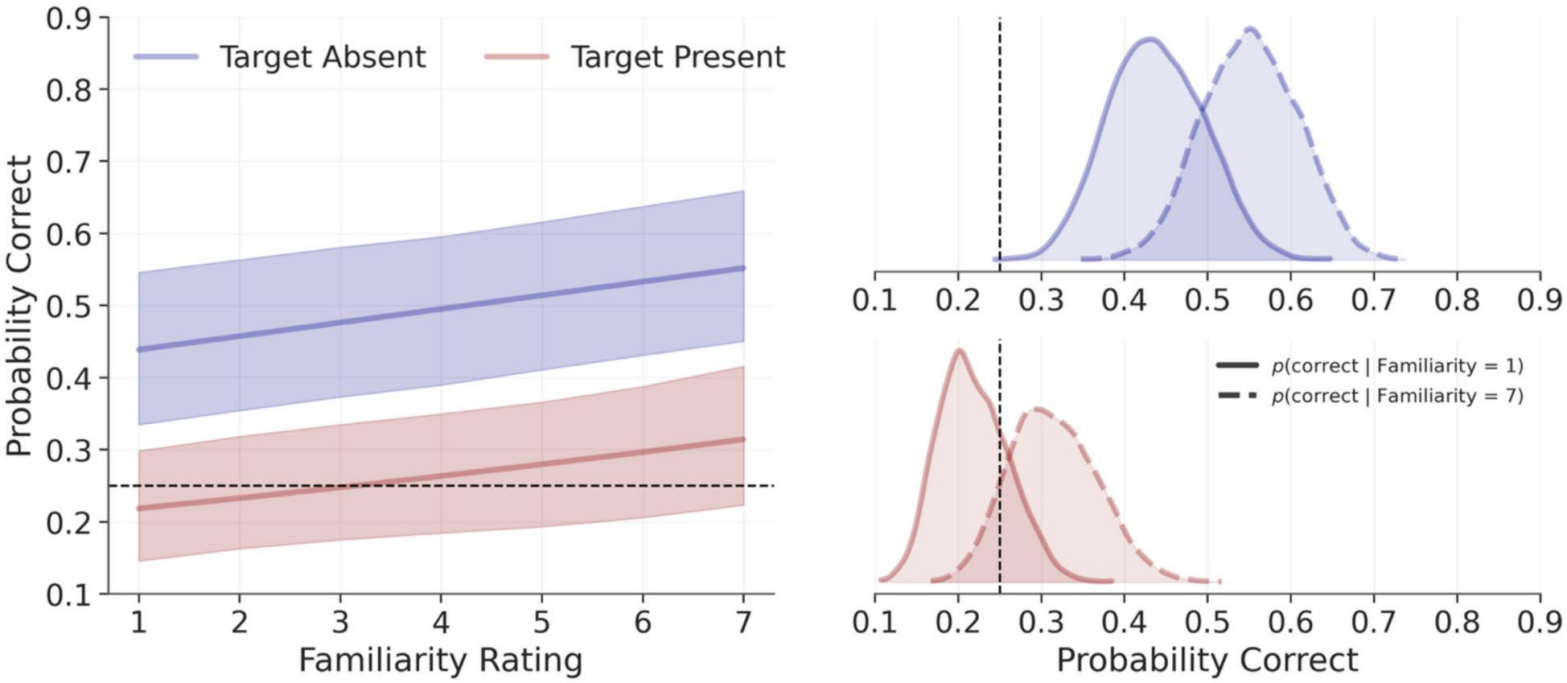
Model predictions for the influence of familiarity on accuracy in Experiment 4*.* The left panel depicts model predictions, where shaded areas represent 94% credible intervals, with synthetic trials shown in red and real trials shown in blue. The right panel depicts posterior distributions of conditional model predictions, setting familiarity to the lowest (one) and highest (seven) levels. The dashed vertical lines represent accuracies expected by chance

As in Experiments 2 and 3, we examined the effects of familiarity on accuracy as odds (see Fig. 9). For ‘target absent’ trials, there was little evidence of increased familiarity aiding trial accuracy, *OR* = 0.99, 94% CrI [0.94, 1.05], *p*(H > 1) = 41.9%, while for ‘target present’ trials, a one-unit increase in familiarity was associated with a 9% increase in accuracy, *OR* = 1.09, 94% CrI [1.04, 1.14], *p*(H > 1) = 100%.

Finally, we predicted accuracy for both trial types at familiarities of one and seven. For ‘target absent’ trials, low familiarity showed levels above-chance accuracy, *M* = 0.44, 94% CrI [0.33, 0.55], *p*(H > 0.25) = 100%, as did high familiarity, *M* = 0.55, 94% CrI [0.45, 0.66], *p*(H > 0.25) = 100%. For ‘target present’ trials, low familiarity showed accuracy where chance was a credible hypothesis, *M* = 0.22, 94% CrI [0.15, 0.30], *p*(H > 0.25) = 22.9%, as did high familiarity, *M* = 0.31, 94% CrI [0.22, 0.42], *p*(H > 0.25) = 89.5%. For ‘target absent’ trials, a correct response was 1.27 times, 94% CrI [1.11, 1.42], *p*(H > 1) = 100%, as likely with high compared to low familiarity. For ‘target present’ trials, a correct response was 1.45 times, 94% CrI [1.18, 1.74], *p*(H > 1) = 100%, as likely with high compared to low familiarity. Taken together, there was a clear (though modest) benefit of increased familiarity on this task (see Fig. 9).

## General discussion

Across our experiments, we demonstrated that an ‘off the shelf’ AI tool (ChatGPT plus DALL·E) can generate photorealistic images of real, familiar individuals which human observers cannot distinguish from genuine photos. Previous studies have tended to consider only a limited database of photographs (taken from Flickr) and the synthesis of fictional counterparts (created using StyleGAN; e.g. Miller et al., 2023; Nightingale & Farid, 2022). As such, using ChatGPT, we first replicated the ability to synthesise images of this style, again confirming that observers were unable to discriminate between these and real photographs. Further, since our approach allowed us to specify all aspects of each synthesised image, we could closely match our images with original photographs to control for non-face characteristics during judgements.

Next, we demonstrated the versatility and additional potential of ChatGPT. Not only could we generate images of another style (i.e. red carpet publicity photos) but we also synthesised images of familiar identities (which were again indistinguishable to observers). The importance of this distinction cannot be overstated. While realistic Flickr-style images of novel identities may add credibility to online fake profiles (and disinformation more broadly), the ability to produce novel/synthetic images of real people opens up a number of avenues for use and abuse. For instance, creators might generate images of a celebrity endorsing a certain product or political stance, which could influence public opinion of both the identity and the brand/organisation they are portrayed as supporting (e.g. Knoll & Matthes, 2017).

Finally, we investigated one method that could improve detection performance—providing additional images for comparison. When faced with a potentially synthetic image of a celebrity, observers will typically have access to other images of that identity (online), allowing for a direct comparison rather than solely relying on their internal representations. Here, we found that accuracy was improved when participants knew that the additional images were real photographs, in particular when the image under consideration was synthetic. In contrast, if the authenticity of these additional images was also unknown, accuracy on ‘target present’ trials (again, when the synthetic image was present) was around chance levels, highlighting the different challenges posed by these two contexts for the same triptych of images. However, it is worth noting that overall accuracy on Experiment 4 was modest but above chance, demonstrating that the presence of additional images, even when it is unclear as to which (if any) are synthetic, may still benefit performance.

Importantly, we found that familiarity with identities was associated with only modest increases in performance across our tasks. This contrasts with evidence from studies involving deepfake videos (e.g. Nas & de Kleijn, 2024), perhaps due to the additional diagnostic cues available in that medium (e.g. audio quality and synchronisation) that may facilitate detection (Groh et al., 2024). Indeed, such cues might explain why observers are often more accurate in general with synthetic videos than static images (Diel et al., 2024). From a more theoretical perspective, we know that familiarity leads to substantial improvements in recognition (e.g. Kramer et al., 2018). However, greater familiarity does not appear to increase sensitivity to subtle visual alterations in a person’s face, and may even reduce it, perhaps because those deviations still fit within our internal identity representations (Brédart & Devue, 2006; Ge et al., 2003). Therefore, the modest improvements found here suggest that the detection of subtle image artefacts or deviations in likeness may show little benefit from familiarity. Indeed, increased familiarity appears to elevate perceived likeness across all images of a person (Ritchie et al., 2018), implying a broad tolerance for variation that, in the context of synthetic image detection, may counteract any expected familiarity advantage. Future research should investigate this relationship between familiarity and tolerance more directly.

Although familiarity in the current work provided only modest performance benefits, it may nonetheless alter the information that participants rely on when evaluating faces to determine their authenticity. Detection of synthetic faces depicting unfamiliar people is likely to be guided by low-level image cues (e.g. lighting inconsistencies, skin texture, or surface irregularities) whereas judgments about familiar faces may involve higher-level, identity-based expectations regarding shape and expression consistency, for instance. Further, familiarity increases the reliance on internal facial features (e.g. Ellis et al., 1979) and this may produce a shift in focus when detecting synthetic images also, although this has yet to be studied. Taken together, familiarity may change how people approach the task of synthetic image detection, even though it does not substantially improve performance.

Previous work has identified the composition of the training dataset as an important factor when synthesising novel identities. If White face photographs are overrepresented during training then the algorithm (e.g. StyleGAN2) produces more realistic White synthetic faces (Miller et al., 2023; Nightingale & Farid, 2022). Here, the composition of ChatGPT/DALL·E’s training corpus is unknown. However, we can assume that these datasets contained larger numbers of images of more famous celebrities. As a result, ChatGPT will likely be better able to generate new images resembling those better-known identities. This is because ChatGPT’s ability to synthesise a new face that closely resembles the one provided is likely determined by its exposure to similar looking faces (with none more similar than the celebrity themselves) during training. Indeed, anecdotally, this was evident during the initial stages of our exploration of the tool. Future work could therefore consider what is presumably a continuum of fame for celebrities that may predict (via their online prevalence) the realism of synthetic images that ChatGPT is capable of generating.

In addition to each synthetic image’s realism/resemblance, we also suspect that viewing conditions will play an important role when determining whether an image is computer-generated or not. In the current work, we took care to present face images at an acceptable size for inspection (and prevented the use of mobile phones). However, the public will likely view online content at smaller scales, making any useful signs of synthesis (e.g. incongruent lighting; Miller et al., 2023) potentially undetectable. Further study will therefore need to consider detection performance in more ecologically valid viewing conditions, e.g. on mobile devices or with smaller/poorer quality images.

Although we found that familiarity with an identity failed to prevent erroneously believing a synthetic image to be real, there were clear individual differences in performance across all tasks. Recent studies have begun to investigate whether super-recognisers (i.e. individuals with superior face recognition abilities) may be better able to detect deepfakes. While such a group showed no advantage when shown deepfake videos (Ramon et al., 2024), evidence suggests super-recognisers may outperform typical observers when tasked with detecting digitally manipulated (Davis et al., 2025) and AI-synthesised face images (Dunn et al., 2025; Gray et al., 2025), although studies have yet to consider deepfakes involving familiar faces. As such, this represents an important avenue for future investigation.

In sum, the present work demonstrates ChatGPT’s ability to generate synthetic images of both novel and familiar faces which are indistinguishable from real photographs to most human observers. Since both familiarity with, and reference images of, a particular identity produced only limited benefits, researchers will need to explore alternative solutions as a matter of urgency. In time, we might find that automated systems will match or surpass human performance in detecting these deepfakes. However, at least for the foreseeable future, the veracity of content will be left for viewers to determine for themselves and, as such, we should make this search for solutions a priority.

## Open practices statement

The raw data, analysis code, and ChatGPT-generated stimuli are available at the Open Science Framework (https://osf.io/fmuh5). Additional information can also be found in the online supplementary materials. Real photographs from Experiment 1 have previously been made available online (Nightingale & Farid, 2022), while copyright permissions prevent us from sharing the real photographs of celebrities used in Experiments 2–4 (although these are accessible via Google Images). The experiments presented here were not preregistered.

## Supplementary Information

Supplementary file 1.

## Data Availability

The raw data, analysis code, and ChatGPT-generated stimuli are available at the Open Science Framework: https://osf.io/fmuh5
