# Supplementary material for: AI-generated images of familiar faces are indistinguishable from real photographs
Source: Cogn Res Princ Implic. 2025 Oct 14;10:70. doi: 10.1186/s41235-025-00683-w (PMC12521686; doi:10.1186/s41235-025-00683-w)
Supplement: Supplementary file 1 [file 41235_2025_683_MOESM1_ESM.docx]

**Supplementary Materials: AI-generated images of familiar faces are indistinguishable from real photographs**

Robin S. S. Kramer, Alex L. Jones^,^ Daniel Fitousi and Jeremy J. Tree

**Supplemental methods**

**Participant data exclusions**

During stimulus creation, it became evident that displaying face images very small onscreen made the task of distinguishing real photos from synthetic images even more difficult (if not impossible). Therefore, to avoid this issue, we prevented participants from completing experiments using their mobile phones through settings in both Gorilla (where the experiments were created and hosted) and Prolific (where participants were recruited). In addition, we asked participants what type of device they were using during the collection of demographic information before each task and rejected those who selected ‘mobile phone’ at that stage. Finally, Gorilla automatically collects information on the size of the display on which the experiment is being completed, and this allowed us to confirm that no participants had used mobile phones. Table S1 summarises participant exclusions across all experiments.

**Table S1** Participant data exclusions. A summary of the reasons why participants were excluded for each experiment

| Reason | Exp 1 | Exp 2 | Exp 3 | Exp 4 |
| --- | --- | --- | --- | --- |
| Did not complete all trials | 1 | 0 | 0 | 0 |
| Completed study on mobile phone | 0 | 0 | 0 | 0 |
| Failed one or more attention check trials | 27 | 22 | 13 | 16 |
| Gave the same response for all experimental trials | 2 | 4 | 0 | 0 |
| Total exclusions | 30 | 26 | 13 | 16 |
| Retained sample | 110 | 115 | 127 | 120 |

ChatGPT settings and prompts

Throughout stimulus creation with ChatGPT, each image was generated in a separate ‘conversation’ to guarantee independent outputs. In addition, ChatGPT’s ‘memory’ option was turned off, as was the ‘improve the model for everyone’ option.

*Experiment 1 prompt:*

Create a new image of a completely fictional adult. Carefully analyse this reference image and generate a photorealistic image that precisely replicates all observed visual details — including skin texture, pores, wrinkles, blemishes, scattered or stray hairs, hairline, facial hair, aging marks, lighting, background, depth of field, lens artifacts, sharpness, colour grading, tonal balance, and grain — preserving the photographic style and quality exactly. The only intended change is replacing this subject’s face with a new, completely fictional person of the same gender and ethnicity. Do not smooth, idealise, or modify any other elements; retain all imperfections, surface textures, and visual realism exactly as in the reference. The output image should be square.

*Experiment 2 prompt:*

This is a fictional person called Alex. I want you to generate a new, highly realistic AI image of Alex at a red carpet or media publicity event. The image should strongly resemble a candid paparazzi photo taken with flash photography.

Character Details:

- Do not replicate the pose in the reference image. Use a clearly different head angle and tilt.

Stylistic Requirements for Realism:

- Emphasise flash lighting on the subject without darkening the background — keep the ambient background light brightness, tone, and saturation identical to the reference image. Match the exposure levels and overall visibility of background elements.
- Include visible skin texture (fine lines, pores), slightly uneven tones, and subtle imperfections for realism.
- Add a small amount of digital grain or film-style noise to break up the overly clean digital look.
- Incorporate mild chromatic aberration or colour fringing on high-contrast edges.
- Simulate shallow depth of field with a slightly blurred background.
- Sharpen details around the eyes, teeth, and clothing edges — common in high-res paparazzi shots.
- Add realistic catchlights in the eyes from flash.
- Keep the background identical to the provided reference image.

Overall Image Quality:

- Match the colour temperature of the reference image.
- Match the brightness and luminance of the reference image.
- Replicate the degree of natural blur found in the reference image. Do not enhance sharpness beyond what’s present in the original. Maintain realistic lens softness on edges and skin.
- Preserve the natural compression artifacts or softness seen in the reference image. Don’t upscale or artificially clean the image beyond what’s visible in the original.

The final image should be square format.

**Adjustment of stimuli using GIMP**

Using GIMP image editor ([www.gimp.org](http://www.gimp.org)), only general image settings (temperature, saturation, brightness, contrast, blur) were adjusted for our generated stimuli (see Fig. S1). Crucially, no changes were made to the synthetic images beyond these overall adjustments, and no changes were made to the real photographs.

**
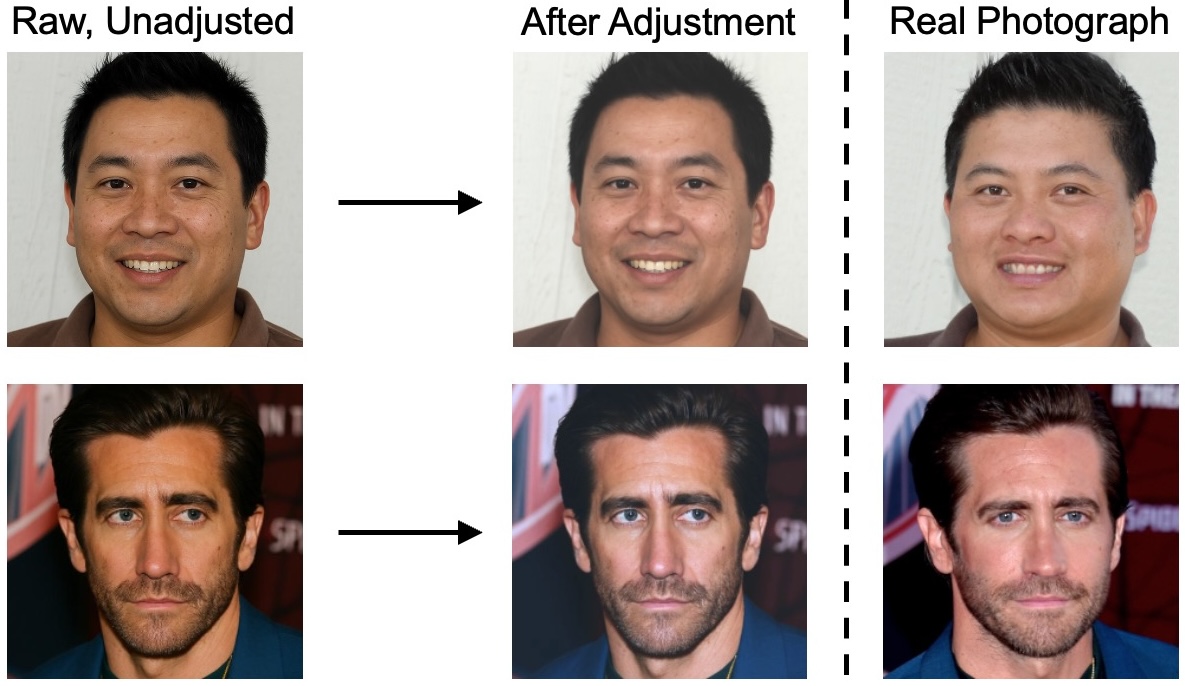
**

**Fig. S1** Example stimuli before and after adjustment using GIMP*.* Examples are provided from Experiment 1 (top row) and Experiments 2-4 (bottom row). Image attribution for the real photo of Jake Gyllenhaal: Toglenn (cropped). The photograph is from Wikimedia Commons (2025) (https://commons.wikimedia.org/)

**Attention check trials**

*Experiment 1:*

Four additional real photographs from the image set of Nightingale and Farid (2022) were selected (and so did not appear in the test set), and these were used to produce four ‘matched’ synthetic images as attention checks (following the same process as for the experimental stimuli). These synthetic images were then altered using GIMP image editor (www.gimp.org) to display obvious distortions/artefacts, so that they were clearly not real photographs (see Fig. S2).


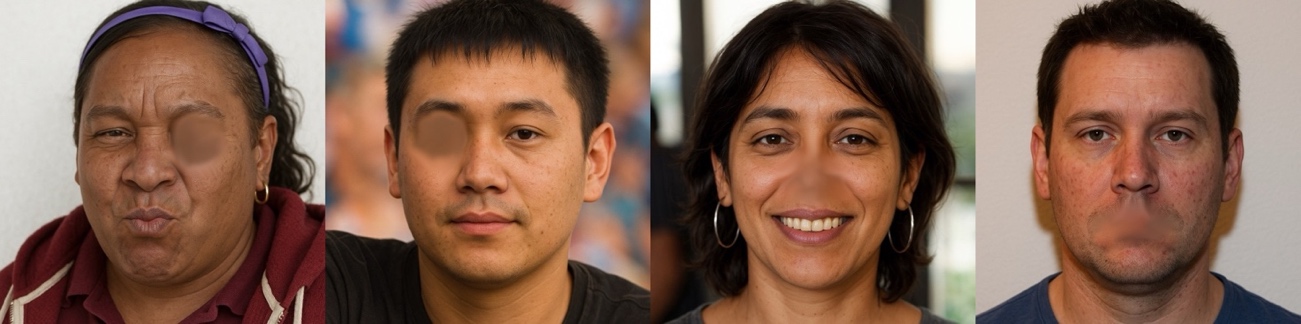


**Fig. S2** Attention check trial images for Experiment 1

*Experiments 2-4:*

Images of four additional celebrities (who did not appear in the test set) were selected, and these were used to produce four ‘matched’ synthetic images as attention checks (following the same process as for the experimental stimuli). These synthetic images were then altered using GIMP image editor as in Experiment 1 (see Fig. S3).


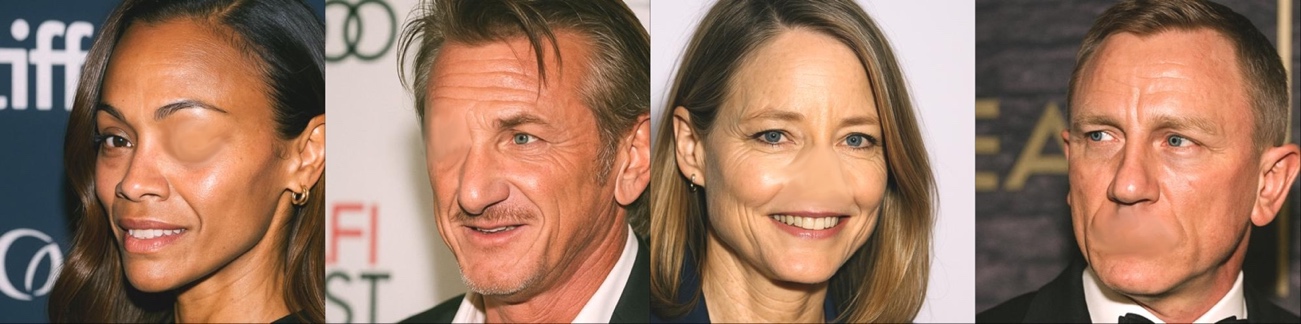


**Fig. S3** Attention check trial images for Experiments 2-4

**Supplemental results**

Raw familiarity data prior to modelling

Readers may be interested in visualising the raw data prior to modelling to give a sense of the patterns we might expect the models to produce (as a ‘sanity check’). To this end, we provide simple plots of trends seen with familiarity ratings, calculated from the raw data (collapsing across all participants and identities).

*Experiment 2:*

We calculated the proportion of correct responses for each level of familiarity. As Figure S4 illustrates, performance on the task was generally poor and close to chance levels (0.5). Further, an increase in familiarity with celebrities failed to provide much improvement overall. Separate inspection of each trial type suggests that higher levels of familiarity tended to produce more ‘real’ responses, resulting in increased accuracy with ‘real photograph’ trials but decreasing accuracy with ‘synthetic image’ trials.


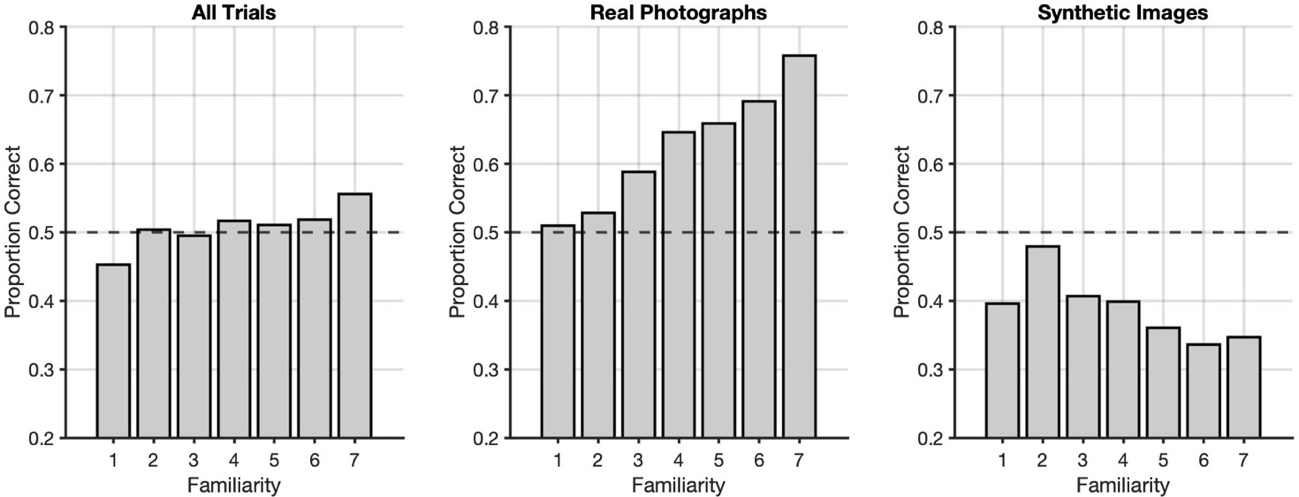


**Fig. S4** The proportion of correct responses, across all participants and identities, for each level of familiarity in Experiment 2*.* The dashed line represents chance performance

*Experiment 3:*

We carried out the same process as above. Here, Figure S5 illustrates that overall performance on the task was a little above chance levels (0.5). Further, an increase in familiarity with celebrities seemed to provide some improvement. Separate inspection of each trial type suggests that higher levels of familiarity tended to increase accuracy with ‘real photograph’ trials while having little effect on accuracy with ‘synthetic image’ trials.


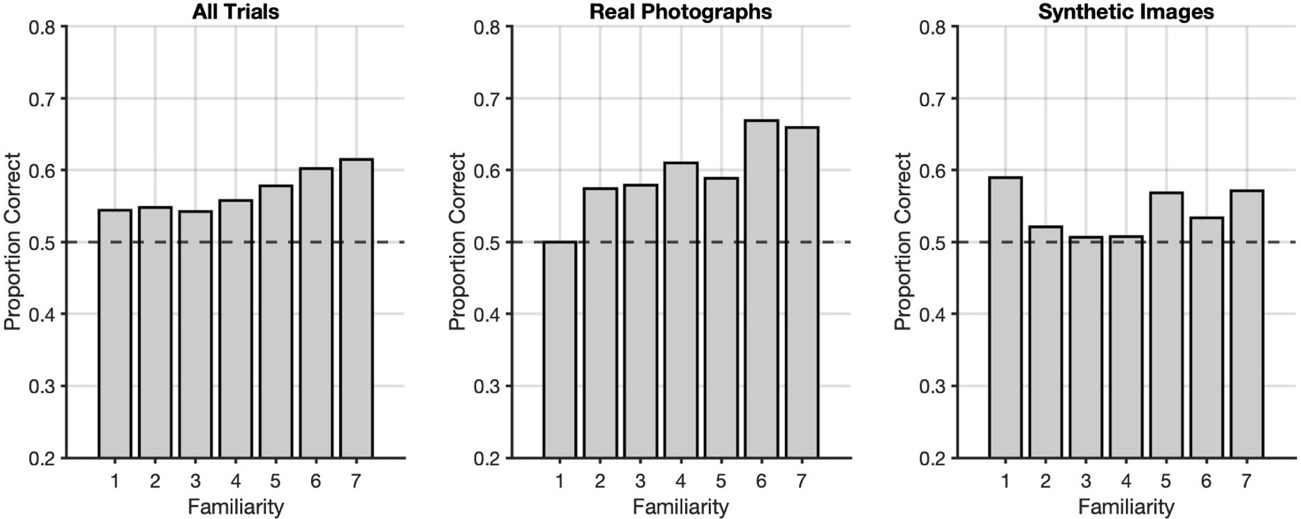


**Fig. S5** The proportion of correct responses, across all participants and identities, for each level of familiarity in Experiment 3*.* The dashed line represents chance performance

Prevalence of lineup response outcomes

In Experiment 4, the target (i.e., the synthetic image) was either present or absent in the lineup. Since participants could identify one of the three images as synthetic *or* none of them, this meant that five response outcomes were possible:

1. Hits – the synthetic image was present *and* correctly chosen
2. Misidentifications – the synthetic image was present *but* the wrong image was chosen
3. Misses – the synthetic image was present *but* none were chosen
4. Correct rejections – the synthetic image was absent *and* none were chosen
5. False alarms – the synthetic image was absent *but* an image was chosen

Figure S6 summarises the prevalence of these response outcomes for each trial type.


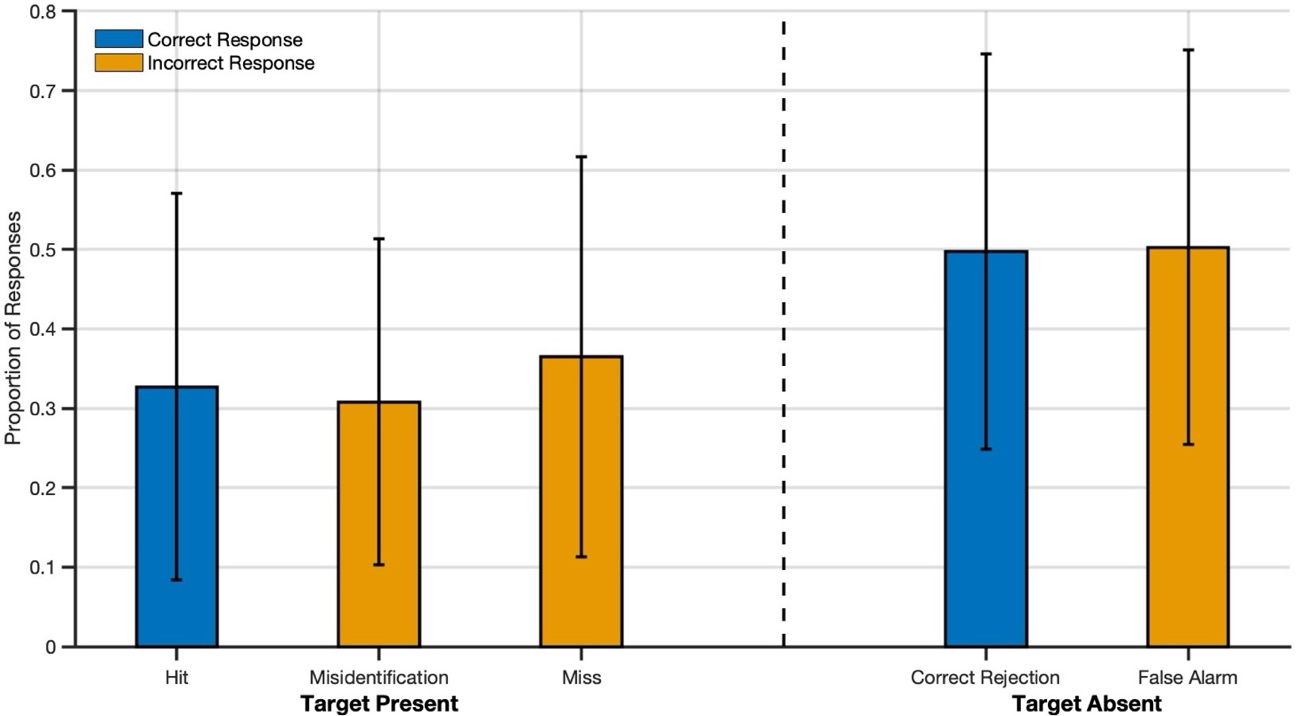


**Fig. S6** The mean proportion of each response outcome within trial type for Experiment 4*.* Error bars represent standard deviations

**References**

Nightingale, S. J., & Farid, H. (2022). AI-synthesized faces are indistinguishable from real faces and more trustworthy. *Proceedings of the National Academy of Sciences, 119*(8), e2120481119.
